# Supplementary material for: HIVprotI: an integrated web based platform for prediction and design of HIV proteins inhibitors
Source: J Cheminform. 2018 Mar 9;10:12. doi: 10.1186/s13321-018-0266-y (PMC5845081; doi:10.1186/s13321-018-0266-y)
Supplement: Supplementary file 2 — Additional file 2. Source code of HIVProtI web server. [file 13321_2018_266_MOESM2_ESM.zip › HIVprotI_Source-code/hivprotI/weka-3-6-6/doc/weka/classifiers/functions/SMO.BinarySMO.html]

SMO.BinarySMO


---


|  |  |  |
| --- | --- | --- |
| **Overview** \| **Package** \| **Class** \| **Tree** \| **Deprecated** \| **Index** \| **Help** \| **Weka's home** | |  |
| **PREV CLASS**   **NEXT CLASS** | **FRAMES**    **NO FRAMES**     **All Classes** |  |
| SUMMARY: NESTED \| FIELD \| CONSTR \| METHOD | DETAIL: FIELD \| CONSTR \| METHOD |  |


---


## weka.classifiers.functions Class SMO.BinarySMO

```
java.lang.Object
  weka.classifiers.functions.SMO.BinarySMO
```

**All Implemented Interfaces:**: java.io.Serializable

**Enclosing class:**: SMO

---

``` public class SMO.BinarySMO extends java.lang.Object implements java.io.Serializable ```

Class for building a binary support vector machine.

**See Also:**: Serialized Form

---

**Constructor Summary**

`SMO.BinarySMO()`


| **Method Summary** | |
| --- | --- |
| `Kernel` | `getKernel()`             Returns the kernel to use |
| `java.lang.String` | `getRevision()`             Returns the revision string. |
| `void` | `setKernel(Kernel value)`             sets the kernel to use |
| `double` | `SVMOutput(int index, Instance inst)`             Computes SVM output for given instance. |
| `java.lang.String` | `toString()`             Prints out the classifier. |

**Methods inherited from class java.lang.Object**

`equals, getClass, hashCode, notify, notifyAll, wait, wait, wait`

**Constructor Detail**

### SMO.BinarySMO

```
public SMO.BinarySMO()
```


**Method Detail**

### setKernel

```
public void setKernel(Kernel value)
```

:   sets the kernel to use

    :   **Parameters:**: `value` - the kernel to use

---


### getKernel

```
public Kernel getKernel()
```

:   Returns the kernel to use

    :   **Returns:**: the current kernel

---


### SVMOutput

```
public double SVMOutput(int index,
                        Instance inst)
                 throws java.lang.Exception
```

:   Computes SVM output for given instance.

    :   **Parameters:**: `index` - the instance for which output is to be computed: `inst` - the instance **Returns:**: the output of the SVM for the given instance **Throws:**: `java.lang.Exception` - in case of an error

---


### toString

```
public java.lang.String toString()
```

:   Prints out the classifier.

    :   **Overrides:**: `toString` in class `java.lang.Object`
    :   **Returns:**: a description of the classifier as a string

---


### getRevision

```
public java.lang.String getRevision()
```

:   Returns the revision string.

    :   **Returns:**: the revision


---


|  |  |  |
| --- | --- | --- |
| **Overview** \| **Package** \| **Class** \| **Tree** \| **Deprecated** \| **Index** \| **Help** \| **Weka's home** | |  |
| **PREV CLASS**   **NEXT CLASS** | **FRAMES**    **NO FRAMES**     **All Classes** |  |
| SUMMARY: NESTED \| FIELD \| CONSTR \| METHOD | DETAIL: FIELD \| CONSTR \| METHOD |  |


---
